# Friend leukemia virus integration 1 is a predictor of poor prognosis of breast cancer and promotes metastasis and cancer stem cell properties of breast cancer cells

**DOI:** 10.1002/cam4.1589

**Published:** 2018-06-04

**Authors:** Xu Yan, Yu Yu, Lingyu Li, Naifei Chen, Wei Song, Hua He, Jie Dong, Xiangliang Liu, Jiuwei Cui

**Affiliations:** ^1^ Pathological Diagnostic Center The First Hospital of Jilin University Changchun Jilin China; ^2^ Cancer Center The First Hospital of Jilin University Changchun Jilin China

**Keywords:** breast carcinoma, epithelial‐mesenchymal transition, Friend leukemia virus integration 1, invasiveness, stemness

## Abstract

Breast cancer is the most common cancer in women worldwide; despite the developments in diagnosis and therapy, recurrence and metastasis remain the main causes of death among patients with breast cancer. This study aimed to identify a promising biomarker for this disease. The study clarified (1) the association between Friend leukemia virus integration 1 (FLI‐1) and various molecular subtypes and (2) the prognostic value of FLI‐1 in breast cancer. To the best of our knowledge, this study is the first to report that FLI‐1 is a predictor of poor prognosis in patients with breast cancer and overexpressed in the triple negative breast cancer (TNBC) subtype. To further verify the effect of FLI‐1 in promoting the metastasis of TNBC, we performed a series of functional experiments in vitro and orthotopic xenograft experiments in the mammary fat pad of nude mice. FLI‐1, as a transcription factor, bound to the promoters of key EMT‐related genes (*CDH1* and *VIM*), and regulated their expressions at the transcriptional level, thus induced epithelial‐mesenchymal transition (EMT). The overexpression of FLI‐1 significantly upregulated the expression of mesenchymal markers. After the modulation of FLI‐1, the changes in mammary stem cell markers (ALDH1A1 and CD133) and the capacity to form mammospheres were consistent with those of the EMT‐related markers. The orthotopic xenograft models further confirmed that the attenuation of stem cell traits after silencing FLI‐1 decreased the ability of tumorigenesis. These results indicate that FLI‐1 is a useful predictor of poor prognosis in patients with breast cancer. Furthermore, the preliminary exploration of metastatic mechanism in the patients with TNBC will provide a potential target to treat breast cancer in the near future.

## 1 INTRODUCTION

Breast cancer is the most common cancer among women worldwide, accounting for approximately 20% of all cancer cases.^1^ Breast cancer represents a highly heterogeneous disease.^2^ Although certain progress has been made in diagnosis and therapy, drug resistance, recurrence, and metastasis are still the main causes of death among patients with breast cancer, which is the second leading cause of cancer‐related mortality in women.^1^ Triple‐negative breast cancer (TNBC) is the most aggressive subtype of breast cancer associated with a high risk of early recurrence, distant metastasis, and worst prognosis. Due to lack of estrogen, progesterone, and human epidermal growth factor 2 (HER2) receptors, the use of cytotoxic agents remains the mainstream measure. However, the responses are not long‐lasting and drug resistance frequently occurs. Therefore, there is a need for early detection and more effective targeted therapies.^3^, ^4^

Friend leukemia virus integration 1 (FLI‐1), a member of E26 transformation‐specific (Ets) transcription factor family, was first identified in the F‐MuLV‐induced mouse erythroleukemia cells by Ben‐David.^5^ FLI‐1 is preferentially expressed in the hematopoietic and vascular endothelial cells and plays an important role in normal cell function and malignant transformation.^6^, ^7^ It has been widely accepted as an oncogene contributing to various malignancies, such as malignant hematological diseases and some solid tumors, including breast cancer.^8^, ^9^, ^10^, ^11^, ^12^, ^13^, ^14^ In our previous study, we showed that the expression of FLI‐1 upregulated in breast cancer tissues compared with that in the nontumor adjacent tissues. Furthermore, the preliminary results indicated that it might be related to apoptosis and metastasis.^15^ Herein, we used tissue microarrays and widened the number of breast cancer samples to (1) further clarify the association between FLI‐1 and various molecular subtypes and (2) evaluate the prognostic value of FLI‐1 in the patients with breast cancer. Based on the immunostaining analysis results, we further performed a series of experiments in vitro, including the wound‐healing assay, transwell assays, colony‐forming assay, and mammosphere‐formation assay. Furthermore, we orthotopically injected the breast cancer cells into the mammary gland of nude mice to explore the molecular mechanism of recurrence and metastasis in the TNBC cells. We expected to identify a promising molecular driving factor, which can be used as a novel potential target to improve prognosis in patients with breast cancer.

## 2 MATERIALS AND METHODS

### 2.1 Immunohistochemistry

The tissue microarray contained 140 breast cancer samples, with clinical characteristics and prognosis information (follow‐up time from 3.3 to 11 years), purchased from the Outdo Biotech Company (HBreD140Su04, Shanghai, China). All of the samples chosen to make the tissue microarray chip were reviewed retrospectively and diagnosed according to the 2012 World Health Organization (WHO) Classification of Tumors of the Breast. Staging was according to the 7th edition of American Joint Committee on Cancer (AJCC 7th) staging manual. The microarray included only the AJCC stage from I to III, as surgery is not recommended for patients with breast cancer in the late stage per the most recent version of NCCN Clinical Practice Guidelines in Oncology (NCCN^®^), available on the NCCN Web site at NCCN.org. The collection of these samples was approved by the ethics committee of the Taizhou Hospital, Zhejiang Province, and all enrolled patients had signed an informed consent. The immunohistochemical analysis was carried out in the Pathological Diagnostic Center, The First Hospital of Jilin University. The mouse monoclonal antibody to FLI‐1 (no dilution, ZM0108; ZSGB‐BIO, Beijing, China) and rabbit polyclonal antibody to aldehyde dehydrogenase 1 family member A1 (ALDH1A1; 1:500) (Cat. no. PA5‐32127; Thermo Fisher Scientific, NY, USA) were used in this study for immunohistochemical staining according to the protocol recommended by the manufacturers. The tissue microarray and the mice tumors sections were deparaffinized, rehydrated, and baked for 1 hour at 65°C. After the retrieval of antigen with ethylenediaminetetraacetic acid (EDTA; pH 9.0, 1:50) (MVS‐0098/0099; Maxim, Fuzhou, China) for 2.5 minutes at 120°C, the endogenous peroxidase was blocked with 3% hydrogen peroxide for 10 minutes at 25°C. After being blocked with 5% bovine serum albumin (BSA) for 2 hours at 25°C, the sections were incubated with FLI‐1 or ALDH1A1 at 37°C for 2 hours, followed by incubation with the MaxVision^™^ HRP Mouse/Rabbit Kit (KIT5020; Maxim, Fuzhou, China) for 15 minutes at 25°C. The incubation time of the DAB Plus Kit (DAB2031; Maxim, Fuzhou, China) depended on the staining intensity. Before the evaluation, the cell nuclei were counterstained with hematoxylin. The immunostaining results were semi‐quantified and scored by two experienced pathologists independently without clinical information. The immunostaining evaluation was performed as previously described.^16^ The median value 4 was identified as an optimal cutoff value for the FLI‐1 expression score: high expression group (final score ≥4) and low expression group (final score <4).

### 2.2 Cell cultures

The human breast cancer cell lines MDA‐MB‐231 and MCF‐7, and human embryonic kidney cell line HEK293 were obtained from the American Type Culture Collection (ATCC). The cells, except for the mammosphere‐formation assay, were cultured in Dulbecco's modified Eagle's medium (DMEM) (Thermo Scientific HyClone, Beijing, China) supplemented with 10% fetal bovine serum (FBS) (Gibco, Gaithersburg, MD, USA), and 1% penicillin and streptomycin (Thermo Scientific HyClone) at 37°C with 5% carbon dioxide (CO_2_) in a humidified atmosphere.

### 2.3 Plasmids construction and lenti‐virus packing

Two short hairpin RNA (shRNA) oligonucleotides (shFLI‐1 1#: 5′‐CGTCATGTTCTGGTTTGAGAT‐3′; shFLI‐1 2#: 5′‐GCACAAACGATCAGTAAGAAT‐3′) targeting FLI‐1 were designed and corresponding pWSLV‐shFLI‐1‐puro‐nanolucGFP plasmids were synthesized by Viewsolid Biotech, Beijing, China. The vector plasmid was provided by Viewsolid Biotech. The full length of FLI‐1 cDNA was amplified and subcloned into the pWPXLd expression vector. Lentiviral supernatant was collected after transfection with shFLI‐1 or expression plasmids along with the lentivirus packaging plasmids psPAX2 and pMD2.G at 48 and 72 hours after transfection. By centrifugation, filtration, purification with polyethylene glycol 8000 (PEG‐8000), and recentrifugation, the lentivirus containing target gene was obtained and used to construct stable cell lines. The stable cell lines were cultured in the presence of 1 μg/mL puromycin (Invitrogen, Carlsbad, CA, USA) for another week. Green fluorescent protein (GFP) was used to assess the transfection efficiency. All the plasmids used in this study were confirmed through sequencing by Comate Biosciences Co., Ltd., Changchun, China.

### 2.4 Small interference RNA transfection

To evaluate the role of FLI‐1 in maintaining the malignant phenotypes in breast cancer, we designed and synthesized two small interference RNA (siRNA) sequences (siFLI‐1 1# and 2#), with si*Luc* (siRNA‐targeting luciferase gene, which is not present in humans) as a negative control (detailed information pertaining to the siRNA sequences could been found in^15^).

The cells were seeded and cultured in a 6‐well plate at 70%‐80% confluency for transfection 24 hours, such as RNA extraction, and 30%‐40% confluency for transfection 48 hours, such as Western blot analysis. The Opti‐MEM medium containing the appropriate amount of transfection agents and siRNA was added into the culture medium. Four to six hours after transfection, the medium was replaced with fresh medium, and the cells were cultured until the endpoint.

### 2.5 Western blot analysis

The expression of FLI‐1 (ab133485, Abcam), E‐cadherin (ab40772, Abcam), β‐catenin (#8480, CST), N‐cadherin (610920, BD bioscience), vimentin (550513, BD bioscience), ALDH1A1 (PA5‐32127, Thermo), and CD133 (MB0160, Bioworld) was determined by Western blotting. Briefly, the cells after transfection were lysed using the radioimmunoprecipitation assay (RIPA) buffer (Lot #71728155; MultiSciences, Hangzhou City, China) and 10 mmol/L phenylmethylsulfonyl fluoride. The homogenate was centrifuged at 12 000 *g* at 4°C for 10 minutes to obtain the supernatant. The content of protein in the supernatant was measured using the BCA Kit (P0010; Beyotime, Nanjing, China). The protein samples (20‐50 μg/lane) were subjected to sodium dodecyl sulfate‐polyacrylamide gel electrophoresis (SDS‐PAGE) with 10%‐12% acrylamide gels and then transferred onto polyvinylidene fluoride (PVDF) membranes. The expression of protein was detected using the indicated antibodies, and then visualized by enhanced chemiluminescence (ECL) (Cat. no. 10200; NCM Biotech, Suzhou, China).

### 2.6 RNA extraction and quantitative real‐time PCR assay

The total RNA from the breast cancer cells was isolated using the TRIzol reagent (Invitrogen) and reverse transcribed into cDNA using the Transcriptor First Strand cDNA Synthesis Kit (Cat. no. 04896866001; Roche, Basel, Switzerland) according to the protocol of the manufacturer.

The quantitative real‐time PCR analysis was performed in an Applied Biosystems 7300 (Bio‐Rad) using the SYBR Green qPCR SuperMix. β‐Actin was used as the internal control. Primers used were listed in Table S1. The relative expression of genes was calculated by the 2^−ΔΔ^ CT method, and each cDNA sample was analyzed thrice.

### 2.7 Luciferase reporter assay

The pGL4‐*CDH1*‐promoter (−1499 to +135) and pGL4‐*VIM*‐promoter (−1499 to +100) luciferase reporter plasmids were presented by Dr. Jingxin Feng (Northeast Normal University, Changchun, China).^17^ The HEK293 cells were seeded in a 12‐well plate (1 × 10^5^ cells/well). After incubation for 24 hours, the cells were co‐transfected with FLI‐1 overexpression plasmid (pW‐FLI‐1, 1 μg) or empty plasmid (pWPLXd, 1 μg), with pGL4‐*CDH1*‐promoter or pGL4‐*VIM*‐promoter luciferase reporter plasmid (*CDH1*‐promoter or *VIM*‐promoter, 500 ng) and *Renilla* luciferase normalization control plasmid 100 ng using the transfection reagent Lipofectamine^™^ 2000 (Invitrogen, Carlsbad, CA, USA). After 48 hours of transfection, the cell lysate was used to measure the activity of luciferase using the Dual‐Luciferase Reporter Assay System (Promega, Wisconsin, USA) according to the instruction of the manufacturer.

### 2.8 Wound‐healing assay

The cells were seeded in a 6‐well plate, and before transfection, the plates were wounded with a sterile pipette tip and photographed. Twenty‐four hours after transfection, the medium was replenished, the plates were photographed again, and the distance of cell migration was measured under an inverted microscope. The cell culture medium contained only 2% FBS to avoid the overproliferation of tumor cells.

### 2.9 Transwell invasion and migration assays

For the transwell invasion assay, the MDA‐MB‐231 cells of siFLI‐1 1# and siLuc at a density of 1 × 10^4^ cells/well were seeded into the top chamber of a transwell insert (#3422, 8.0 μm pore size; Corning Inc., NY, USA) and 15% FBS‐containing medium was added into the bottom chamber. After incubation at 37°C in 5% CO_2_ for 36‐48 hours, the cells that remained on the upper surface of the membrane were gently removed. The cells that migrated through the pores were fixed with 4% paraformaldehyde, stained with 0.1% crystal violet, and photographed with an Olympus BX51 and Olympus DP20. Five fields were randomly selected, counted, and analyzed using the GraphPad Prism 7.0 software.

For the transwell migration assay, the conditions were similar to that of the migration assay, except for the differences of cell density, incubation time, and lack of matrigel in the inserts.

### 2.10 Colony formation assay

The stable shFLI‐1 NC and shFLI‐1 1# MDA‐MB‐231 cells were seeded in a 6‐well plate at a density of 200 cells/well and cultured for 10‐14 days. The medium was replenished every 3 days. The plates were fixed with 4% paraformaldehyde and stained with 0.1% crystal violet. The number of colonies was counted and recorded. The experiments were performed in triplicate and repeated thrice.

### 2.11 Mammosphere‐formation assay

The mammosphere‐formation assay has been adapted to quantify stem cell activity and self‐renewal. According the detailed protocol recommended by Shaw et al*,* 18 the stable transfection cell lines (shFLI‐1 NC and 1# of MDA‐MB‐231 cells) were seeded into a 6‐well plate with ultralow attachment surface (#3471, Corning Inc., Corning, NY, USA) and cultured in DMEM/F12 (Thermo Scientific HyClone, Beijing, China) supplemented with 10 ng/mL recombinant human epidermal growth factor (EGF) (Cat. no. AF‐100‐15; Peprotech, Rochy Hill, NJ, USA), 10 ng/mL recombinant human basic fibroblast growth factor (bFGF) (Cat. no. 100‐18B; Peprotech), 2% B27^™^ supplement 50 ×  (LOT. 1860141; Gibco, Gaithersburg, MD, USA). Seven days after incubation, the plates were gently moved and observed to count the number of mammospheres formed. The mammospheres (magnification, 400 × ) of diameter >50 μm were judged as an effective mammosphere.

### 2.12 Orthotopic xenograft experiments with nude mice

In compliance with the animal management rules of the Chinese Ministry of Health, the animal experiment was approved by the Institutional Animal Care and Use Committee of the Northeast Normal University. Twenty‐four‐wk‐old female BALB/c nude mice were purchased from Hua Fu Kang Bioscience Co., Ltd., Beijing, China and maintained under specific pathogen‐free conditions. Following a 1‐wk recovery period, the mice were randomly divided into two groups (n = 10 per group). The stable cell lines (MDA‐MB231 shFLI‐1 NC and shFLI‐1 1#) were harvested and suspended in DMEM. A 100‐μL mixture, containing 3 × 10^6^ cells, DMEM and matrigel (6:1), was injected into the fat pad of the fourth mammary gland in the lower right abdomen of each mouse.^19^, ^20^ The mice were monitored for tumor development, and 30 days after the injection of cancer cells, the mice were euthanized. The orthotopic tumors, lungs, and brains were removed, measured, weighed, and fixed in 4% neutral paraformaldehyde for further histopathological analysis.

### 2.13 Statistical analysis

All statistical analyses of tissue microarray were performed using the SPSS version 23.0 software (SPSS Inc., Chicago, IL, USA). The association between FLI‐1 and clinicopathological characteristics was analyzed by the chi‐squared test. The differences of FLI‐1 expression in different subtypes were analyzed by Mann‐Whitney *U* test (A threshold of *P *<* *.005 was defined as statistically significant after the correction by Bonferroni's method.). The survival data were evaluated by the univariate and multivariate *Cox* regression analyses. The OS and DFS curves were plotted by the Kaplan‐Meier method and analyzed with the log‐rank test. The data of other experiments were analyzed using the GraphPad Prism software version 7.0 (GraphPad Software, San Diego, CA, USA). The one‐way analysis of variance (ANOVA) was used to identify the differences between various groups. A threshold of *P *<* *.05 was defined as statistically significant.

## 3 RESULTS

### 3.1 High FLI‐1 expression correlates with poor prognosis of patients with breast cancer

**Figure 1:**
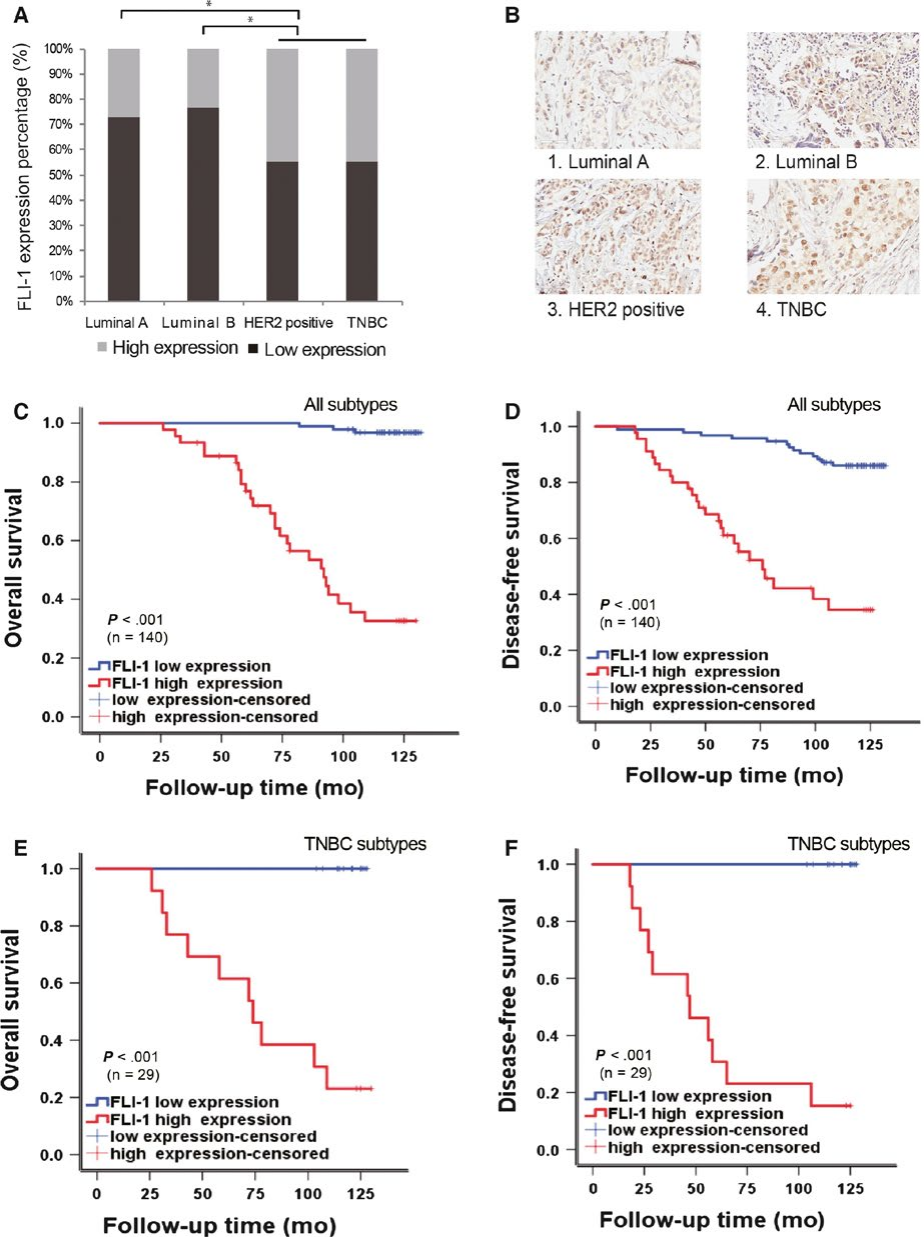
FLI‐1 expression differs from different molecular subtypes and high expression of FLI‐1 positively correlates with poor OS and DFS in the patients with breast cancer. A, Differential expressions of FLI‐1 in the four molecular subtypes. *P <* *.005. B, Representative images of immunohistochemical staining with the FLI‐1 antibody of different molecular subtypes (magnification, 400 × ). C, The Kaplan‐Meier overall survival curves of all 140 patients with breast cancer (Log‐rank *P* value < .001). D, The Kaplan‐Meier disease‐free survival curves of all 140 patients with breast cancer (Log‐rank *P* value <.001). E, The Kaplan‐Meier overall survival curves of the patients with TNBC subtype breast cancer (Log‐rank *P* value <.001). F, The Kaplan‐Meier disease‐free survival curves of the patients with TNBC subtype breast cancer (Log‐rank *P* value <.001). These curves were stratified by high and low expression of FLI‐1

In our previous study, we revealed that the expression of FLI‐1 is significantly upregulated in the breast cancer tissues compared with that in the corresponding adjacent nontumor tissues (paired tumor and adjacent tissues of 53 cases).^15^ To further evaluate the relevance of clinical molecular subtypes and clarify the prognostic value of FLI‐1 in breast cancer, patient‐derived tissue microarray was carried out using 140 breast cancer samples. The clinicopathological characteristics of patients with breast cancer are summarized in Table 1. The tissue microarray slide was stained with FLI‐1 antibody and scored (summarized in Table 2). In the angiosarcoma tissue, FLI‐1 was strongly positive for the specificity and sensitivity of antibody tests (representative image is presented in Supporting Information). By the analyses of Mann‐Whitney *U* test after correction, among the four subtypes, FLI‐1 were upregulated in the HER2 positive and triple‐negative subtypes compared with Luminal A/B subtypes (HER2 positive vs Luminal A/B subtype, *P *<* *.001 and TNBC vs Luminal A/B subtype, *P *<* *.001, respectively] (Figure 1A). Figure 1B is the most representative image of different molecular subtypes for FLI‐1 staining (magnification, 400 × ). Through the correlation analysis between FLI‐1 and the clinicopathological features of breast cancer (as shown in Table 2), we found that the expression of FLI‐1 was higher in patients with breast cancer with lymph node metastasis compared with that in patients without metastasis (*P *=* *.02). Moreover, the expression of FLI‐1 was lower during the early stage and higher during the advanced stage of breast cancer (*P *=* *.007). No significant association was observed between FLI‐1 expression and age (*P *=* *.268) and HER2 status (*P *=* *.625) in the single factor statistical analysis. Overall, these results showed that FLI‐1 positively correlated with lymph node metastasis and clinical stage. Furthermore, it had relevance with molecular subtypes, indicating that FLI‐1 might be related to poor outcomes in patients with breast cancer.

**Table 1:** Clinicopathological characteristics of the breast cancer tissue microarray and the expression of FLI‐1

Characteristic	Number of cases (%)
Age (y)	
<60	93 (66.4)
≥60	47 (33.6)
Lymph node metastasis	
No	79 (56.4)
Yes	61 (43.6)
HER2 (Fluorescence in situ Hybridization, FISH)	
Negative	115 (82.1)
Positive	25 (17.9)
AJCC stage	
I	33 (23.6)
II	61 (43.6)
III	46 (32.9)
Molecular subtype	
Luminal A	85 (60.7)
Luminal B	17 (12.1)
HER2 positive	9 (6.4)
TNBC	29 (20.7)
Expression of FLI‐1	
Low expression group	95 (67.9)
High expression group	45 (32.1)

**Table 2:** Correlation between FLI‐1 expression and the clinicopathological characteristics in breast cancer

Characteristics	FLI‐1		*P* value
	Low expression group	High expression group	
Age (y)			
<60	66	27	.268
≥60	29	18	
Lymph node metastasis			
No	60	19	.020
Yes	35	26	
HER2 (FISH)			
Negative	77	38	.625
Positive	18	7	
AJCC stage			
I	25	8	.007
II	47	14	
III	23	23	
Molecular subtype			
Luminal A	62	23	.112
Luminal B	13	4	
HER2 positive	5	4	
TNBC	16	13	

Therefore, we used the *Cox* regression analysis of overall survival (OS) and disease‐free survival (DFS) to evaluate whether FLI‐1 can serve as a prognostic factor. The univariate *Cox* analysis demonstrated that the OS of patients with high expression of FLI‐1 was relatively shorter (*P *<* *.001) (Table 3), which was similar to lymph node metastasis (*P *=* *.005) and advanced stage (*P *=* *.008). However, in the multivariate *Cox* regression analysis, only FLI‐1 showed a definite correlation with the OS of patients with breast cancer. Similar results were obtained from the univariate and multivariate *Cox* regression analyses of DFS (Table 4). Further, FLI‐1 was closely related to the recurrence and death among patients with breast cancer (*P *<* *.001). The Kaplan‐Meier log‐rank survival curves demonstrated that the OS and DFS of all patients with breast cancer with high expression of FLI‐1 were significantly shorter than those in patients with low expression Median survival time (Figure 1C,D). Median OS of all patients with high expression of FLI‐1 was 92 months (95% CI: 72.024‐111.976 months) and 76 months (95% CI: 56.682‐95.318 months), respectively. Median OS and DFS of all patients with low expression of FLI‐1 was not reached for patients by the data cutoff date. Moreover, we extracted TNBC subtype, which is the most aggressive subtype without effective target therapy, from all patients with breast cancer and generated the Kaplan‐Meier survival curves. The curves demonstrated that the patients with TNBC with high expression of FLI‐1 had a poor prognosis (Figure 1E,F). These results indicated that the overexpression of FLI‐1 in the patients with breast cancer correlated with poor survival. Furthermore, FLI‐1 can be an independent prognostic factor for breast cancer.

**Table 3:** Univariate and multivariate *Cox* regression analyses of various prognostic parameters in the OS of patients with breast cancer

Prognostic parameter	Univariate analysis				Multivariate analysis			
	HR	*P*	95.0 CI		HR	*P*	95.0 CI	
			Lower limit	Upper limit			Lower limit	Upper limit
FLI‐1 (high vs low)	35.181	.000	10.548	117.347	30.486	.000	8.679	107.086
Age (≥60 y vs <60 y)	2.21	.034	1.063	4.574	2.714	.075	1.135	6.491
Lymph node metastasis (yes vs no)	3.112	.005	1.417	6.838	1.170	.799	0.349	3.920
AJCC stage		.008				.728		
II stage vs I stage	1.735	.409	0.470	6.409	1.861	.426	0.404	8.584
III stage vs I stage	4.719	.013	1.382	16.114	1.680	.547	0.311	9.074
Molecular subtype vs luminal A type		.011				.167		
Luminal B type	1.303	.685	0.363	4.669	1.151	.841	0.292	4.531
HER2 positive type	4.843	.003	1.680	13.960	2.823	.075	0.900	8.857
TNBC type	2.911	.014	1.236	6.854	2.599	.061	0.959	7.047

**Table 4:** Univariate and multivariate *Cox* regression analyses of various prognostic parameters in the DFS of patients with breast cancer

Prognostic parameter	Univariate analysis				Multivariate analysis			
	HR	*P*	95.0 CI		HR	*P*	95.0 CI	
			Lower limit	Upper limit			Lower limit	Upper limit
FLI‐1 (high vs low	7.984	.000	4.008	15.905	5.339	.000	2.569	11.096
Age (≥60 y vs <60 y)	1.151	.681	0.588	2.251	1.370	.397	0.662	2.834
Lymph node metastasis (yes vs no)	3.362	.001	1.694	6.670	1.150	.790	0.410	3.223
AJCC stage		.000				.144		
II stage vs I stage	.909	.867	0.297	2.779	0.934	.912	0.279	3.132
III stage vs I stage	4.724	.002	1.805	1.805	2.531	.176	0.660	9.075
Molecular subtype vs luminal A type		.009				.189		
Luminal B type	1.513	.419	0.554	4.131	1.383	.534	0.497	3.848
HER2 positive type	4.699	.001	1.831	12.062	2.921	.033	1.088	7.838
TNBC type	2.271	.036	1.054	4.895	1.681	.205	0.752	3.757

### 3.2 Modulation of FLI‐1 expression influences the expression of EMT‐related proteins in the breast cancer cells

**Figure 2:**
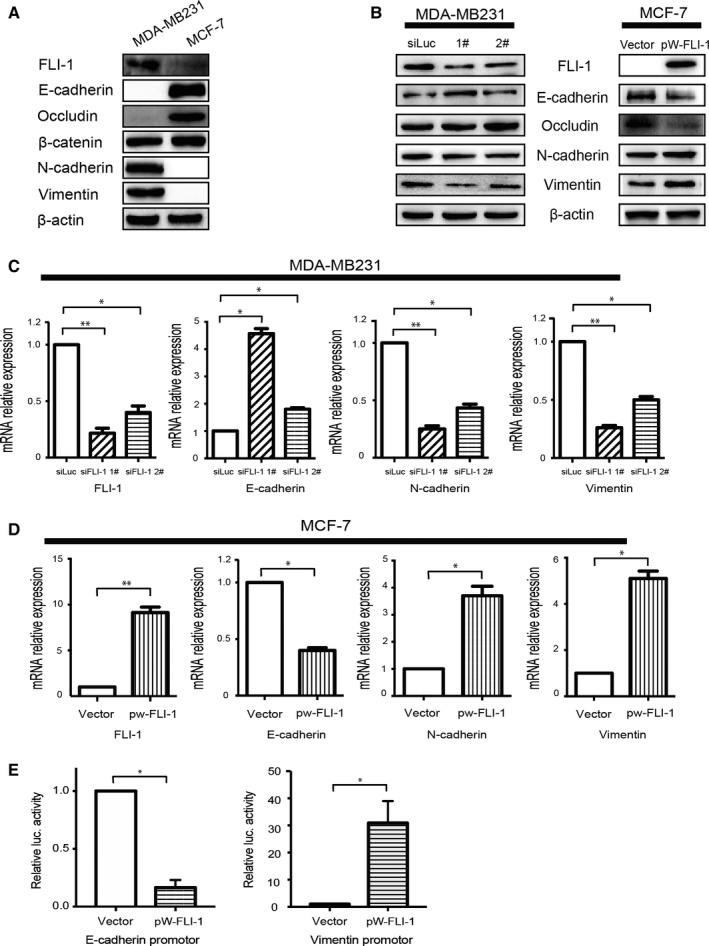
Modulation of FLI‐1 expression influences the expression of EMT‐related proteins in the breast cancer cells. A, The difference in the expression of EMT‐related proteins in the MDA‐MB231 and MCF‐7 cell lines. B, The changes in the expression of EMT‐related proteins (N‐cadherin, vimentin, E‐cadherin, and Occludin) after the knockdown of FLI‐1 expression in MDA‐MB231 and forced expression of FLI‐1 in the MCF‐7 cells. C, The changes in the expression of EMT‐related genes (N‐cadherin, Vimentin, and E‐cadherin) after the modulation of FLI‐1 expression in the MDA‐MB231 and MCF‐7 cells. Each bar represents mean ± standard deviation (SD). *P <* *.05. **P <* *.005. D, The overexpression of FLI‐1 increased the activity *VIM* (Vimentin) promoter luciferase activity, but distinctly decreased the activity of *CDH1* (E‐cadherin) promoter luciferase. Each bar represents mean ± SD. *P <* *.05

We previously reported that FLI‐1 was distinctly overexpressed in the MDA‐MB231 cell line, whereas its expression decreased in the MCF‐7 cell line.^15^ The latter is a luminal A type breast cancer cell line, and the former has been the most widely accepted TNBC subtype with mesenchymal properties.^21^, ^22^ Studies have demonstrated that tumor cells can eventually enter the invasion‐metastasis cascade, a prerequisite is the acquisition of mesenchymal properties—epithelial‐mesenchymal transition (EMT).^4^, ^23^ Combined with the tissue analysis results and the previous finding that FLI‐1 activated the Rho GTPase pathway,^15^ a pathway closely related to the EMT process,^24^ elucidated the potential association between FLI‐1 and the EMT program. To verify the hypothesis, we compared the difference in the expression of EMT‐related proteins in the MDA‐MB231 and MCF‐7 cell lines. As expected, in the MDA‐MB231 cells, FLI‐1 and mesenchymal markers (N‐cadherin and vimentin) were overexpressed, but the expression of epithelial markers (E‐cadherin and occludin) decreased. We obtained the converse results in the MCF‐7 cells (Figure 2A).

To further clarify the association between FLI‐1 and the EMT process, we evaluated changes in the EMT‐related markers by modulating the expression of FLI‐1. The knockdown of FLI‐1 expression in MDA‐MB231 downregulated the expression of N‐cadherin and vimentin, whereas it upregulated the expression of epithelial markers. After the forced expression of FLI‐1 in the MCF‐7 cells, the expression of mesenchymal markers was upregulated, whereas the expression of E‐cadherin and occludin decreased (Figure 2B). Similar results were obtained at the mRNA level measured by real‐time PCR (Figure 2C). The above results elucidated the correlation between FLI‐1 and EMT.

During the EMT process, some transcription factors, such as epithelial‐mesenchymal transition‐transcription factors (EMT‐TFs), are essential^25^; FLI‐1 is a transcription factor. To elucidate the role of FLI‐1 as an EMT‐TF in the EMT process, the dual‐luciferase reporter gene assay was carried out. The results revealed that the overexpression of FLI‐1 increased the activity of *VIM* (Vimentin) promoter luciferase, but distinctly decreased the activity of *CDH1* (E‐cadherin) promoter luciferase (Figure 2D). These data indicated that FLI‐1 modulated key EMT‐related markers by influencing the activities of their promoters at the transcriptional level, thus upregulating the mesenchymal markers and downregulating epithelial markers.

Overall, FLI‐1 can induce the EMT program by regulating the promoters of EMT‐related key genes at the mRNA level in breast cancer cells.

### 3.3 Knockdown of FLI‐1 expression inhibits the invasion and metastasis abilities of breast cancer cells

**Figure 3:**
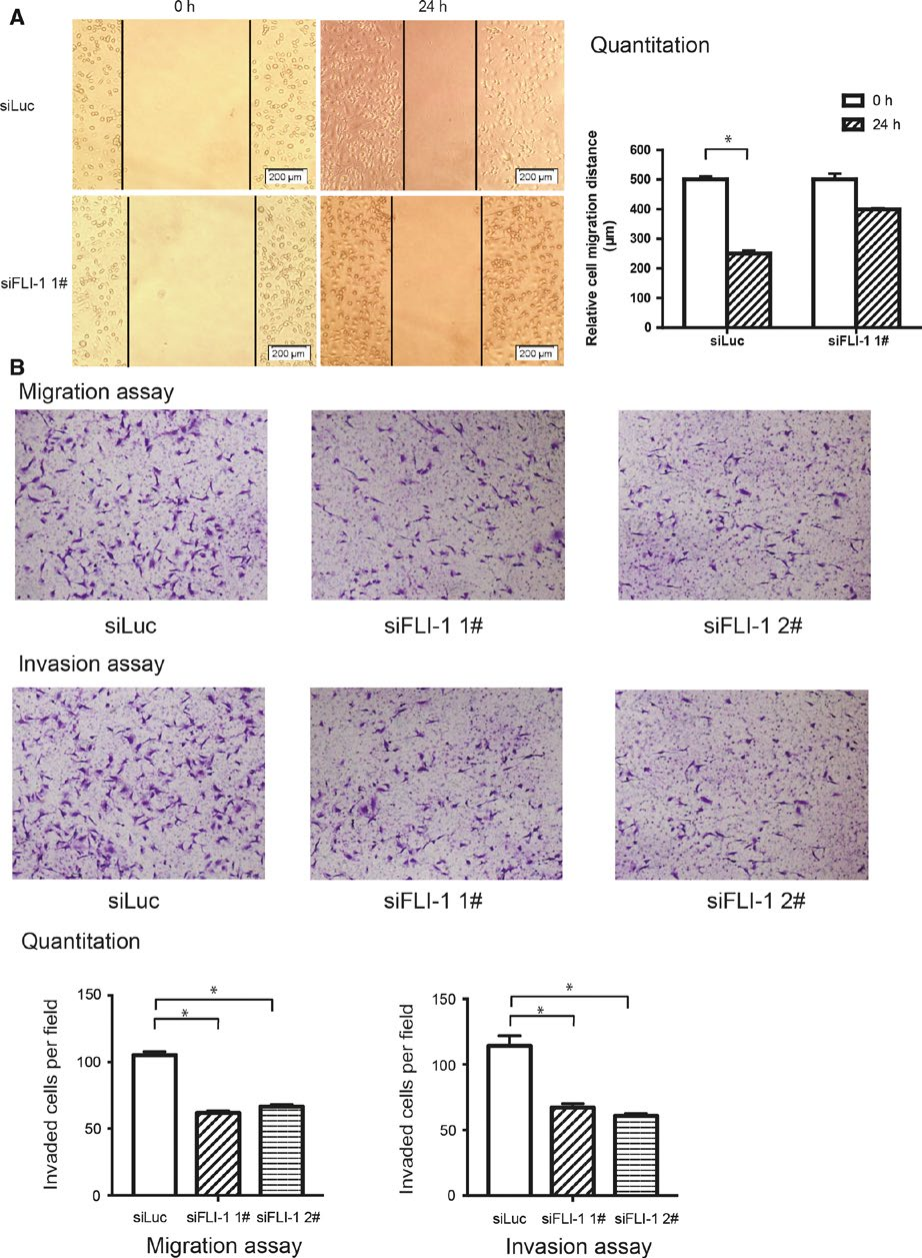
Knockdown of FLI‐1 expression inhibits the invasive and metastatic abilities of the MDA‐MB231 cells. A, The wound‐healing assay demonstrated that silencing FLI‐1 distinctly decreased cell motility. B, The transwell migration and invasion assays demonstrated that the knockdown of FLI‐1 inhibited the invasiveness and metastatic abilities of the cells. Each bar represents mean ± standard deviation. *P <* *.05

During tumor progression, the EMT has been generally accepted, which empowers the migratory and invasive abilities of tumor cells.^3^, ^26^ To further verify whether FLI‐1 can affect the invasiveness and metastasis of breast cancer cells, we performed wound‐healing and transwell assays after the knockdown of FLI‐1. The wound‐healing assay demonstrated that the silencing of FLI‐1 distinctly decreased cell motility compared with that of control group in the MDA‐MB231 cells (Figure 3A). Similarly, the transwell migration and invasion assays also demonstrated that the knockdown of FLI‐1 inhibited the cell invasive and metastatic abilities (Figure 3B).

### 3.4 Knockdown of FLI‐1 suppresses cancer stem cell properties in vitro and inhibits tumorigenesis in vivo

**Figure 4:**
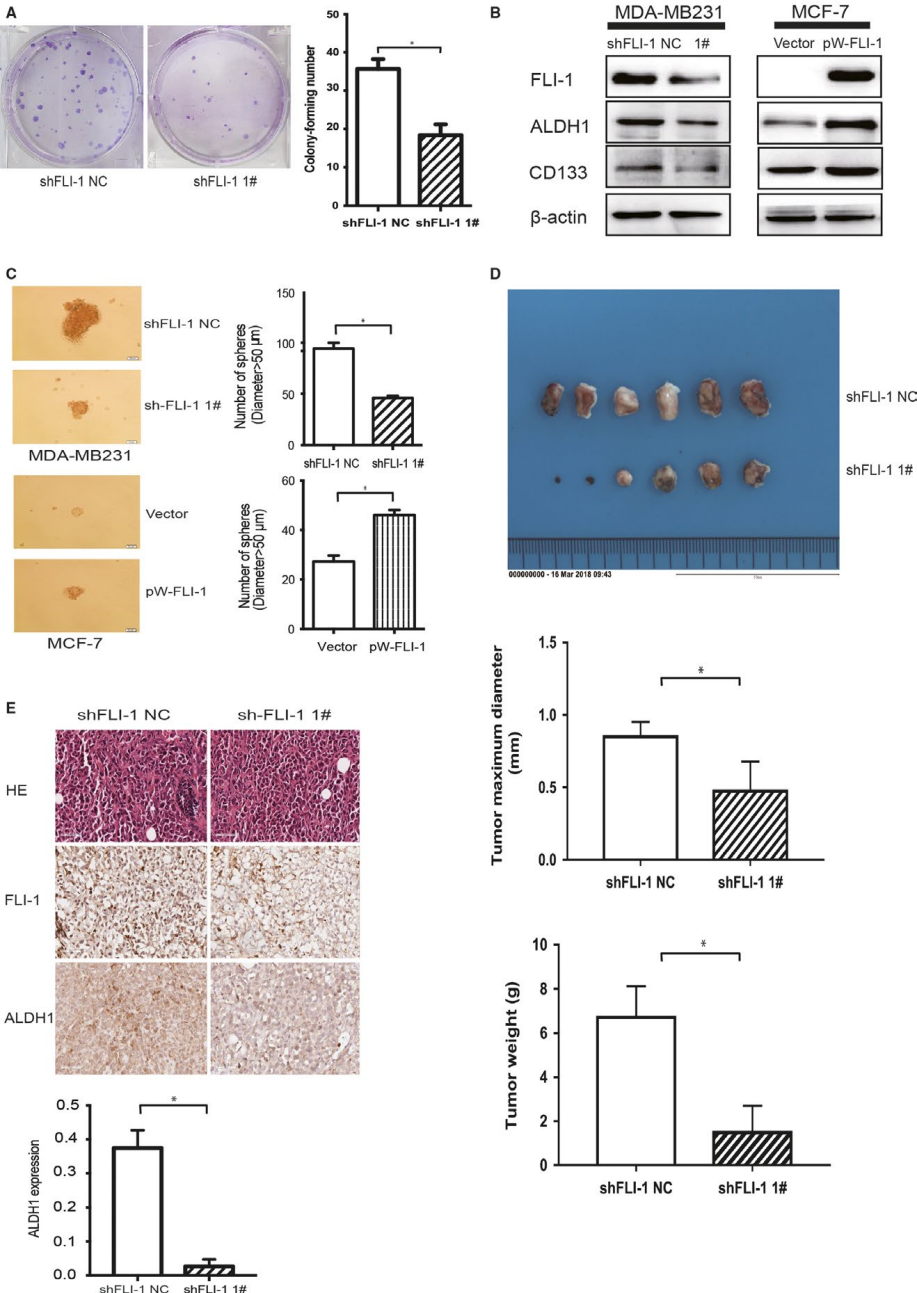
Knockdown of FLI‐1 inhibits clonogenicity and cancer stem cell properties in vitro and suppresses tumorigenesis in vivo. A, The colony‐forming assay demonstrated that the knockdown of FLI‐1 reduced the cell colony‐forming number. B, The knockdown of FLI‐1 decreased the expression of mammary stem cell markers (ALDH1 and CD133) compared with those of the control group. The overexpression of FLI‐1 in the MCF‐7 cells upregulated the expression of stem cell markers. C, In the shFLI‐1 group of the MDA‐MB231 cells, the sphere‐forming ability was attenuated. The overexpression of FLI‐1 in the MCF‐7 cells upregulated the sphere‐forming capacity. Each bar represents mean ± standard deviation (SD). *P <* *.05. D, The images of excised tumors 30 days after the orthotopic injection of tumor cells into the mammary gland fat pad. The statistical analyses of tumor weight and tumor diameter between the two groups. Each bar represents mean ± SD. *P <* *.05. E, The representative images of tumor sections stained with hematoxylin and eosin, anti‐FLI‐1, and anti‐ALDH1. The statistical analysis of the expression of ALDH1 between the two groups. Each bar represents mean ± SD. *P <* *.05

The EMT is a critical regulator of the CSC phenotype, and studies have reported that the EMT can generate tumor cells, and even normal cells, with CSC‐like properties.^3^, ^27^, ^28^ We had proved that FLI‐1 correlates positively with the EMT, and silencing FLI‐1 inhibited the expression of mesenchymal markers. Therefore, we further investigated the correlation between FLI‐1 and the CSC phenotype of breast cancer cells. The colony‐forming assay indicated that the colony‐forming number significantly decreased in the shFLI‐1 1# group compared with that in the shNC group (Figure 4A). The levels of mammary stem cell markers, aldehyde dehydrogenase 1 (ALDH1) 29, 30, 31 and CD133,^32^, ^33^ were determined by Western blotting. The expression of ALDH1 and CD133 visibly downregulated in the shFLI‐1 group compared with that in the negative control group of the MDA‐MB231 cells. Conversely, in the MCF‐7 cells, the forced expression of FLI‐1 increased the expression of mammary stem cell markers (Figure 4B). In the mammosphere‐formation assay, we found that in the shFLI‐1 group of the MDA‐MB231 cells, the sphere‐forming ability was attenuated. The overexpression of FLI‐1 in the MCF‐7 cells resulted in the upregulation of sphere‐forming capacity (Figure 4C).

In vivo transplantation is the gold standard assay of CSC‐like properties.^18^ By the orthotopic injection of breast cancer cells into the mouse mammary gland, we found that the formation of orthotopic tumors in the shFLI‐1 group mice was significantly delayed than that in the shNC group mice (Figure 4D). Although we failed to observe distant metastasis till the end of the experiment, we found that the expression of ALDH1 positively correlated with that of FLI‐1. The most representative images of HE, FLI‐1, and ALDH1 are presented in Figure 4E. Furthermore, the statistical analysis results of ALDH1 level between the shNC and siFLI‐1 1# groups demonstrated that silencing FLI‐1 distinctly reduced the expression of ALDH1 (Figure 4E).

In brief, FLI‐1 correlated positively with the tumor‐initiating capacity by influencing the CSC‐like properties.

## 4 DISCUSSION

Some ETS factors have already been proved to be dysregulated in breast cancer, but the role of FLI‐1 in breast cancer is still controversial. The mainstream studies, including our previous study, suggest that FLI‐1 inhibits cell apoptosis or promotes tumor progression in breast cancer.^13^, ^14^, ^15^ However, Scheiber et al^34^ presented a contradictory conclusion that reduced expression of FLI‐1 promotes tumor progression. Currently, to the best of our knowledge, no study has elucidated the association between FLI‐1 and the prognosis of breast cancer. In the present study, we focused on the prognostic value of FLI‐1 in patients with breast cancer and its preliminary mechanisms in the TNBC cells.

Based on our previous finding that FLI‐1 is upregulated in the breast cancer tissues, in present study, we carried out tissue microarray on a large number of samples, with thorough clinical characteristics and prognosis (survival, recurrence, and metastasis), to further clarify the prognostic value of FLI‐1 in patients with breast cancer. The immunohistochemical analysis showed that FLI‐1 was significantly related to the clinical late stage, lymph node metastasis, and reduced survival time (OS and DFS). Furthermore, even in the multivariate *Cox* regression analysis, only FLI‐1 expression exhibited a definite correlation with survival time, instead of, the universally accepted, clinical stage, and lymph node metastasis. Therefore, the expression of FLI‐1 will be a novel independent and an extremely useful factor indicating poor prognosis in patients with breast cancer.

From the results of immunohistochemistry, we found that FLI‐1 was overexpressed in the HER2 positive and TNBC subtypes when compared to that in the luminal subtypes of breast cancer. The patients with TNBC constituted approximately 20% of all breast cancers in the present study, which was consistent with the epidemiological studies. However, the number of HER2‐positive patients with breast cancer contained in this microarray was only nine (6.4% of 140 cases), which was significantly lower than that in the epidemiological study.^35^ Therefore, it was essential for us to further verify the overexpression of FLI‐1 in the HER2‐positive subtype. Due to lack of molecular targets and high heterogeneity, different from those of the HER2‐amplified breast cancers, TNBC could not benefit from the progression of recent targeted therapy. Furthermore, cytotoxic chemotherapy, only partially, reduced the recurrence and death risk, making TNBC a challenge requiring more effective targeted therapies.^36^, ^37^ Owing to these reasons, we focused our study on TNBC. Through the statistical analysis of TNBC extracted, we found that even in patients with TNBC, FLI‐1 exhibited a negative correlation with survival. These data strongly indicate that FLI‐1 is a valuable prognostic predictor in patients with breast cancer.

One of the major characteristics of malignant carcinomas is metastasis, which is the main cause of breast cancer‐related deaths. As a prerequisite of metastasis, tumor cells lose the epithelial phenotype and acquire mesenchymal properties. The activation of Rho GTPase, that is, RhoA, Rac1, and Cdc42, is often involved in the migratory and metastatic biological behaviors of tumor cells.^24^ FLI‐1 can activate RhoA and Rac1, which correlates with breast cancer metastasis.^15^ The MDA‐MB231 cell line with a high expression of FLI‐1 has already been proved to possess mesenchymal properties and exhibit high expression of EMT‐associated genes.^21^, ^22^ These findings indicate that FLI‐1 might be related to the EMT program in the MDA‐MB231 cells. After comparing the differences in the EMT‐related markers between the MDA‐MB231 and MCF‐7 cell lines, we initially deduced that FLI‐1 might have a positive relationship with the EMT process. Through the knockdown of FLI‐1 in the MDA‐MB231 cell line and forced expression of FLI‐1 in the MCF‐7 cell line, we further confirmed the association between FLI‐1 and the EMT program. As a transcription factor, FLI‐1 can play its biological functions by recognizing and directly binding to sequence‐specific DNA of many promoters and enhancers at the transcriptional level, resulting in the activation or suppression of the corresponding genes. In the present study, similar to that of an EMT‐TFs, FLI‐1 induced the EMT, at least partially, via the transcriptional inhibition of *CDH1* and transcriptional activation of *VIM*.

The EMT and CSC‐like properties are the malignant phenotypes of cancers,^26^, ^38^ the former contributes to invasion, migration, and metastasis; and the latter endows tumor cells with the capacity of tumorigenesis, recurrence, and therapy resistance.^3^, ^27^, ^39^, ^40^ All these factors are responsible for the poor prognosis of breast cancer. Studies have elucidated that there is a close association between the CSC‐like traits and EMT program.^3^, ^27^, ^40^ EMT endows the mammary epithelial cells and cancer cells with the stem cell properties, including the upregulation of CSC specific cell‐surface markers (ie, elevated expression of ALDH1A1 and CD133 27, 38), which is essential for the initiation and progression of breast cancer.^37^, ^41^ Through in vitro experiments, we demonstrated that FLI‐1 is pivotal for the acquisition and sustenance of CSC‐like properties. To further investigate the role of FLI‐1 in breast cancer tumorigenesis in vivo, orthotopic xenografting of tumors was carried out instead of the conventional subcutaneous or tail intravenous injection.^19^, ^20^ This method is thought to, at least partially, simulate a biologically relevant microenvironment for tumor cells and is a gold standard to evaluate CSC‐like properties.^18^, ^19^ From the histopathological analysis of the harvested orthotopic tumors, we proved that the knockdown of FLI‐1 can decrease the expression of ALDH1 and inhibit tumor growth, which further confirmed the results of the experiments in vitro. We failed to obtain optimal outcomes in terms of metastasis till the end of the experiment. In the future, by reducing the number of tumor cells and extending the feeding time, we hope to obtain the expected result, that is, FLI‐1 can promote the metastasis of breast cancer in vivo.

The results of the present study revealed that FLI‐1 is a novel and an extremely useful predictor for the poor prognosis in patients with breast cancer. The modulation of FLI‐1 affected the mesenchymal characteristics, which was consistent with the changes in CSC‐like phenotype. FLI‐1 can induce EMT by binding to the promoters of EMT‐related key genes (*CDH1* and *VIM*), sustain CSC‐like properties, and influence tumorigenesis in vivo. Targeting FLI‐1 might eliminate the mesenchymal and stem cell traits and would be a novel approach to improve the prognosis of breast cancer.

## CONFLICT OF INTEREST

The authors have no conflict of interest to declare.

## References

[1] Siegel RL , Miller KD , Jemal A . Cancer statistics, 2017. CA Cancer J Clin. 2017;67:7‐30. 28055103 10.3322/caac.21387

[2] Oltmann J , Heselmeyer‐Haddad K , Hernandez LS , et al. Aneuploidy, TP53 mutation, and amplification of MYC correlate with increased intratumor heterogeneity and poor prognosis of breast cancer patients. Genes Chromosom Cancer. 2018;57:165‐175. 29181861 10.1002/gcc.22515 PMC5807164

[3] Lee A , Djamgoz MBA . Triple negative breast cancer: emerging therapeutic modalities and novel combination therapies. Cancer Treat Rev. 2018;62:110‐122. 29202431 10.1016/j.ctrv.2017.11.003

[4] Ignatov A , Eggemann H , Burger E , Ignatov T . Patterns of breast cancer relapse in accordance to biological subtype. J Cancer Res Clin Oncol. 2018. 10.1007/s00432-018-2644-2 PMC11813410 29675790

[5] Ben‐David Y , Giddens EB , Bernstein A . Identification and mapping of a common proviral integration site Fli‐1 in erythroleukemia cells induced by Friend murine leukemia virus. Proc Natl Acad Sci U S A. 1990;87:1332‐1336. 2304901 10.1073/pnas.87.4.1332 PMC53469

[6] Li Y , Luo H , Liu T , Zacksenhaus E , Ben‐David Y . The ets transcription factor Fli‐1 in development, cancer and disease. Oncogene. 2015;34:2022‐2031. 24909161 10.1038/onc.2014.162 PMC5028196

[7] Liu F , Walmsley M , Rodaway A , Patient R . Fli1 acts at the top of the transcriptional network driving blood and endothelial development. Curr Biol. 2008;18:1234‐1240. 18718762 10.1016/j.cub.2008.07.048

[8] Cui JW , Vecchiarelli‐Federico LM , Li YJ , Wang GJ , Ben‐David Y . Continuous Fli‐1 expression plays an essential role in the proliferation and survival of F‐MuLV‐induced erythroleukemia and human erythroleukemia. Leukemia. 2009;23:1311‐1319. 19282832 10.1038/leu.2009.20

[9] Song W , Hu L , Li W , et al. Oncogenic Fli‐1 is a potential prognostic marker for the progression of epithelial ovarian cancer. BMC Cancer. 2014;14:424. 24923303 10.1186/1471-2407-14-424 PMC4089852

[10] Liang X , Shi D , Yun J , et al. Friend leukemia virus integration 1 expression has prognostic significance in nasopharyngeal carcinoma. Transl Oncol. 2014;7:493‐502. 25171891 10.1016/j.tranon.2014.04.015 PMC4202802

[11] Lin SF , Wu CC , Chai CY . Increased FLI‐1 expression is associated with poor prognosis in non‐small cell lung cancers. Appl Immunohistochem Mol Morphol. 2016;24:556‐561. 26317314 10.1097/PAI.0000000000000227

[12] Torlakovic EE , Slipicevic A , Florenes VA , Chibbar R , DeCoteau JF , Bilalovic N . Fli‐1 expression in malignant melanoma. Histol Histopathol. 2008;23:1309‐1314. 18785112 10.14670/HH-23.1309

[13] Mhawech‐Fauceglia P , Herrmann FR , Bshara W , et al. Friend leukaemia integration‐1 expression in malignant and benign tumours: a multiple tumour tissue microarray analysis using polyclonal antibody. J Clin Pathol. 2007;60:694‐700. 16917000 10.1136/jcp.2006.039230 PMC1955051

[14] Sakurai T , Kondoh N , Arai M , et al. Functional roles of Fli‐1, a member of the Ets family of transcription factors, in human breast malignancy. Cancer Sci. 2007;98:1775‐1784. 17727680 10.1111/j.1349-7006.2007.00598.x

[15] Song W , Li W , Li L , et al. Friend leukemia virus integration 1 activates the Rho GTPase pathway and is associated with metastasis in breast cancer. Oncotarget. 2015;6:23764‐23775. 26156017 10.18632/oncotarget.4350 PMC4695150

[16] Pang J , Yan X , Cao H , et al. Knockdown of COPS3 Inhibits Lung Cancer Tumor Growth in Nude Mice by Blocking Cell Cycle Progression. J Cancer. 2017;8:1129‐1136. 28607586 10.7150/jca.16201 PMC5463426

[17] Feng J , Li L , Zhang N , et al. Androgen and AR contribute to breast cancer development and metastasis: an insight of mechanisms. Oncogene. 2017;36:2775‐2790. 27893717 10.1038/onc.2016.432

[18] Shaw FL , Harrison H , Spence K , et al. A detailed mammosphere assay protocol for the quantification of breast stem cell activity. J Mammary Gland Biol Neoplasia. 2012;17:111‐117. 22665270 10.1007/s10911-012-9255-3

[19] Kocaturk B , Versteeg HH . Orthotopic injection of breast cancer cells into the mammary fat pad of mice to study tumor growth. J Vis Exp. 2015. 10.3791/51967 PMC4354624 25742185

[20] Tavera‐Mendoza LE , Brown M . A less invasive method for orthotopic injection of breast cancer cells into the mouse mammary gland. Lab Anim. 2017;51:85‐88. 26994106 10.1177/0023677216640706

[21] Lehmann BD , Bauer JA , Chen X , et al. Identification of human triple‐negative breast cancer subtypes and preclinical models for selection of targeted therapies. J Clin Invest. 2011;121:2750‐2767. 21633166 10.1172/JCI45014 PMC3127435

[22] Andreopoulou E , Schweber SJ , Sparano JA , McDaid HM . Therapies for triple negative breast cancer. Expert Opin Pharmacother. 2015;16:983‐998. 25881743 10.1517/14656566.2015.1032246 PMC5995333

[23] Nieto MA , Huang RY , Jackson RA , Thiery JP . Emt: 2016. Cell. 2016;166:21‐45. 27368099 10.1016/j.cell.2016.06.028

[24] Shibue T , Weinberg RA . EMT, CSCs, and drug resistance: the mechanistic link and clinical implications. Nat Rev Clin Oncol. 2017;14:611‐629. 28397828 10.1038/nrclinonc.2017.44 PMC5720366

[25] Jansen S , Gosens R , Wieland T , Schmidt M . Paving the Rho in cancer metastasis: Rho GTPases and beyond. Pharmacol Ther. 2018;183:1‐21. 28911825 10.1016/j.pharmthera.2017.09.002

[26] Kalluri R , Weinberg RA . The basics of epithelial‐mesenchymal transition. J Clin Invest. 2009;119:1420‐1428. 19487818 10.1172/JCI39104 PMC2689101

[27] Morrison BJ , Schmidt CW , Lakhani SR , Reynolds BA , Lopez JA . Breast cancer stem cells: implications for therapy of breast cancer. Breast Cancer Res. 2008;10:210. 18671830 10.1186/bcr2111 PMC2575525

[28] Mani SA , Guo W , Liao MJ , et al. The epithelial‐mesenchymal transition generates cells with properties of stem cells. Cell. 2008;133:704‐715. 18485877 10.1016/j.cell.2008.03.027 PMC2728032

[29] Santisteban M , Reiman JM , Asiedu MK , et al. Immune‐induced epithelial to mesenchymal transition in vivo generates breast cancer stem cells. Cancer Res. 2009;69:2887‐2895. 19276366 10.1158/0008-5472.CAN-08-3343 PMC2664865

[30] Ablett MP , Singh JK , Clarke RB . Stem cells in breast tumours: are they ready for the clinic? Eur J Cancer. 2012;48:2104‐2116. 22542086 10.1016/j.ejca.2012.03.019

[31] Yang F , Xu J , Tang L , Guan X . Breast cancer stem cell: the roles and therapeutic implications. Cell Mol Life Sci. 2017;74:951‐966. 27530548 10.1007/s00018-016-2334-7 PMC11107600

[32] Ginestier C , Hur MH , Charafe‐Jauffret E , et al. ALDH1 is a marker of normal and malignant human mammary stem cells and a predictor of poor clinical outcome. Cell Stem Cell. 2007;1:555‐567. 18371393 10.1016/j.stem.2007.08.014 PMC2423808

[33] Wright MH , Calcagno AM , Salcido CD , Carlson MD , Ambudkar SV , Varticovski L . Brca1 breast tumors contain distinct CD44 + /CD24‐ and CD133 + cells with cancer stem cell characteristics. Breast Cancer Res. 2008;10:R10. 18241344 10.1186/bcr1855 PMC2374965

[34] Brugnoli F , Grassilli S , Lanuti P , et al. Up‐modulation of PLC‐beta2 reduces the number and malignancy of triple‐negative breast tumor cells with a CD133(+)/EpCAM(+) phenotype: a promising target for preventing progression of TNBC. BMC Cancer. 2017;17:617. 28870198 10.1186/s12885-017-3592-y PMC5584040

[35] Scheiber MN , Watson PM , Rumboldt T , et al. FLI1 expression is correlated with breast cancer cellular growth, migration, and invasion and altered gene expression. Neoplasia. 2014;16:801‐813. 25379017 10.1016/j.neo.2014.08.007 PMC4212256

[36] Lambertini M , Santoro L , Del Mastro L , et al. Reproductive behaviors and risk of developing breast cancer according to tumor subtype: a systematic review and meta‐analysis of epidemiological studies. Cancer Treat Rev. 2016;49:65‐76. 27529149 10.1016/j.ctrv.2016.07.006

[37] Kwa MJ , Adams S . Checkpoint inhibitors in triple‐negative breast cancer (TNBC): where to go from here. Cancer. 2018;124:2086‐2103. 29424936 10.1002/cncr.31272

[38] Hennessy BT , Gonzalez‐Angulo AM , Stemke‐Hale K , et al. Characterization of a naturally occurring breast cancer subset enriched in epithelial‐to‐mesenchymal transition and stem cell characteristics. Cancer Res. 2009;69:4116‐4124. 19435916 10.1158/0008-5472.CAN-08-3441 PMC2737191

[39] Devarajan E , Song YH , Krishnappa S , Alt E . Epithelial‐mesenchymal transition in breast cancer lines is mediated through PDGF‐D released by tissue‐resident stem cells. Int J Cancer. 2012;131:1023‐1031. 22038895 10.1002/ijc.26493

[40] Choi HJ , Park JH , Park M , et al. UTX inhibits EMT‐induced breast CSC properties by epigenetic repression of EMT genes in cooperation with LSD1 and HDAC1. EMBO Rep. 2015;16:1288‐1298. 26303947 10.15252/embr.201540244 PMC4766458

[41] Pindiprolu S , Krishnamurthy PT , Chintamaneni PK . Pharmacological targets of breast cancer stem cells: a review. Naunyn Schmiedebergs Arch Pharmacol. 2018;391:463‐479. 29476201 10.1007/s00210-018-1479-3

