# Supplementary material for: Friend leukemia virus integration 1 is a predictor of poor prognosis of breast cancer and promotes metastasis and cancer stem cell properties of breast cancer cells
Source: Cancer Med. 2018 Jun 4;7(8):3548–60. doi: 10.1002/cam4.1589 (PMC6089157; doi:10.1002/cam4.1589)
Supplement: Supplementary file 1 [file CAM4-7-3548-s001.pdf]

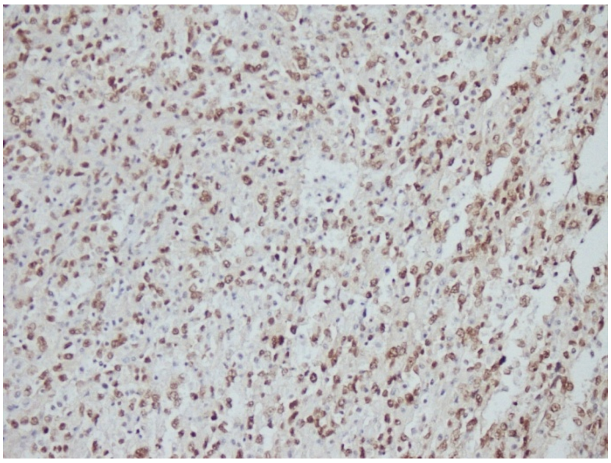

Supplementary Figure E1. FLI-1 was strongly positive in angiosarcoma tissue (Magnification, 200×).

Supplementary Table E1. Primers for real-time quantitative polymerase chain reaction

Name	Sequence 5'-3'
hFLI-1 F	CTGTTGTCACACCTCAGTTAC
hFLI-1 R	CATGTTATTGCCCCAAGCTCCTC
FLI-1-pWPXL F	AGCTTTGTTTAAACATGGACGGGACTATTAAGGA
FLI-1-pWPXL R	GACTAGTCTAGTAGTAGCTGCCTAAGTGTGAA
hE-cadherin F	GACAACAAGCCCGAATT
hE-cadherin R	GGAAACTCTCTCGGTCCA
hN-cadherin F	CGGGTAATCCTCCCAAATCA
hN-cadherin R	CTTTATCCCGGCGTTTCATC
hVimentin F	GAGAACTTTGCCGTTGAAGC
hVimentin R	GCTTCCTGTAGGTGGCAATC
β-actin-F	CAGGTCATCACCATTGGCAATGAGC

β-actin-R	CGGATGTCCACGTCACACTTCATGA
